# Health Tract, No. 228

**Published:** 1865

**Authors:** 


HEALTH TRACT, No. 228.

### POPULAR FALLACIES.

THAT warm air must be impure, and that, consequently, it is hurtful to sleep in a comparatively warm room. A warm room is as easily ventilated as a cool one. The warm air of a close vehicle is less injurious, be it ever so foul, from crowding, than to ride and sit still and feel uncomfortably cold for an hour. The worst that can happen from a crowded conveyance is a fainting spell; while, from sitting even less than an hour in a still, chilly atmosphere, has induced attacks of pneumonia, that is, inflammation of the lungs, which often prove fatal in three or four days. It is always positively injurious to sleep in a close room where water freezes, because such a degree of cold causes the negatively poisonous carbonic acid gas of a sleeping-room to settle near the floor, where it is breathed and rebreathed by the sleeper, and is capable of producing typhoid fevers in a few hours. Hence, there is no advantage, and always danger, especially to weakly persons, in sleeping in an atmosphere colder than the freezing-point.

That it is necessary to the proper and efficient ventilation of a room, even in warm weather, that a window or door should be left open; this is always hazardous to the sick and convalescent. Quite as safe a plan of ventilation, and as efficient, is to keep a lamp or a small fire burning in the fire-place. This creates a draft, and carries bad airs and gases up the chimney.

That out-door exercise before breakfast is healthful. It is never so. And, from the very nature of things, is hurtful, especially to persons of poor health; although the very vigorous may practice it with impunity. In winter the body is easily chilled through and through, unless the stomach has been fortified with a good warm breakfast; and in warm weather, miasmatic and malarious gases and emanations speedily act upon the empty and weak stomach in a way to vitiate the circulation and induce fever and ague, diarrhea, and dysentery; entire families, who have arranged to eat breakfast before leaving the house, and to take supper before sun-down, have had a complete exemption from fever and ague, while the whole community around them was suffering from it, from having neglected these precautions.

That whatever lessens cough is "good" for it, and, if persevered in, will cure it. On the contrary, all coughs are soonest cured by promoting and increasing them; because nature endeavors by the cough to help bring up the phlegm and yellow matter which is in the lungs, as the lungs can not heal while that matter is there. And as it can not be got rid of without coughing, the more coughing there is, the sooner it is got rid of—the sooner are the lungs cleared out for the fuller and freer reception of pure air, which is their natural food. The only remedies which can do any good in coughs are such as loosen the phlegm, and thus less cough is required to bring it up. Those remedies are, warmth, out-door exercise, and any thing which slightly nauseates.

